# Novel biomarkers of preterm brain injury from blood transcriptome in sheep model of intrauterine asphyxia

**DOI:** 10.1038/s41390-024-03224-1

**Published:** 2024-05-31

**Authors:** C. Joakim Ek, Mårten Alkmark, Ana A. Baburamani, Veena G. Supramaniam, Sanjana Sood, Rossella Melchiotti, Emanuele de Rinaldis, Henrik Hagberg, Carina Mallard

**Affiliations:** 1 Centre for Perinatal Medicine and Health, Institutes of Neuroscience and Physiology & Clinical Sciences, Sahlgrenska Academy, Gothenburg University, Gothenburg, Sweden; 2 Centre for the Developing Brain, Department of Perinatal Imaging and Health, School of Biomedical Engineering and Imaging Sciences, King’s Health Partners, St Thomas’ Hospital, London, SE1 7EH UK; 3 Department of Cancer Epidemiology and Population Health, King’s College London, London, UK

## Abstract

**Background:**

Infants born preterm have a higher incidence of neurological deficits. A key step in finding effective treatments is to identify biomarkers that reliably predict outcome.

**Methods:**

Following umbilical cord occlusion (UCO) in pregnant sheep, whole fetal blood RNA was sequenced pre- and post-UCO, brain injury outcome was determined by battery of neuropathology scoring and the transcriptome signature correlated to the degree of brain injury. Additionally, we developed a novel analytical procedure to deduce cell blood composition over time.

**Results:**

Sixty-one genes were identified with significant altered expression after UCO. In pre-UCO blood, the level of three mRNAs (*Trex2*, *Znf280b*, novel miRNA) and in post-UCO, four mRNAs (*Fam184a*, *Angptl2*, novel lincRNA and an unknown protein-coding gene) were associated to brain injury (FDR < 0.01). Several of these mRNAs are related to inflammation and angiogenesis. Pathway analysis highlighted genes playing a role in perinatal death and growth failure. Results also indicate that several leukocyte populations undergo significant changes after UCO.

**Conclusion:**

We have used a whole transcriptomic approach to uncover novel biomarkers in fetal blood that correlate to neuropathology in the preterm sheep brain. The current data forms a basis for future studies to investigate mechanisms of these mRNAs in the injury progression.

**Impact:**

- Trend analysis of genes following asphyxia reveal a group of genes associated with perinatal death and growth failure.
- Several pre-asphyxia transcripts were associated to brain injury severity suggesting genomic susceptibility to injury.
- Several post-asphyxia transcripts were correlated to brain injury severity, thus, serve as potential novel biomarkers of injury outcome.
- Successfully adaptation of cell profiling algorithms suggests significant changes in blood cell composition following asphyxia.

## Introduction

Brain injury in preterm infants is a major clinical problem causing mortality and neurological morbidity.^1^ Approximately 30% of infants born extremely preterm (<28 weeks of gestation) will suffer from cerebral palsy (CP), cognitive impairment or behavioral deficits including autism spectrum disorders and ADHD.^2–5^ Many factors are likely to contribute to preterm brain injury including genetic vulnerability^6^ and adverse intrauterine environment.^7^ In addition, there are various exposures such as inflammation, hyperoxia, cerebral hypoxia-ischemia that are implicated to cause brain lesions in preterm infants.^8,9^

Besides magnesium sulfate neuroprophylaxis,^10^ there are no therapies available which improves neurologic outcome in infants born preterm. In order to develop new interventions there is an urgent need for early reliable biomarkers that can predict outcome in these cases. There are factors that are associated with adverse outcome such as male sex, ethnicity, low level of parental education and low birth weight.^11^ Further, magnetic resonance imaging (MRI) is highly predictive of outcome^12^ but usually performed days-weeks after birth. There is currently a lack of molecular biomarkers that can be detected sufficiently early to allow intervention in the critical phase of injury progression. To date, some biomarkers have been reported^13^ and the field of biomarkers for neonatal hypoxic-ischemic encephalopathy reviewed by Murray in 2019,^14^ however, none of them have shown sufficient predictive power to be implemented in the clinic.

The fetal sheep model is a useful pre-clinical, translational model utilized to gain comprehensive understanding of the development of preterm brain injury by examining both the neuropathology and systemic responses following intrauterine asphyxia.^15,16^ Pre-clinical large animal models, such as fetal sheep preparations, replicate major features of human preterm brain injury and provide access to complex, clinically relevant studies of cerebral blood flow and neuroimaging that are not plausible in smaller laboratory animals.^15,16^ The brain developmental stage of preterm fetal sheep (100 days of gestation, i.e., 0.7 of term gestation in sheep) equates to 24–28 weeks’ preterm infant.^15^ This is the first study to perform comprehensive gene sequencing in the blood over an extended period (7 days) after intrauterine asphyxia in preterm fetal sheep. By deep sequencing fetal blood, we investigate the mRNA gene expression before and following preterm brain injury. Using global gene expression in fetal blood we aimed to generate a comprehensive profile and molecular signatures associated with the development of brain injury in the preterm brain. Such novel data will aid in understanding the pathogenesis of injury and could reveal new therapeutic targets. Most importantly, they could form the basis for the development of early prognostic biomarkers to be used in clinic for early detection of preterm brain injury.

## Material and methods

### Surgery and animal maintenance

Animal experiments were approved by the local Animal Ethics Committee of Gothenburg (Dnr 166-2013 with amendment 2-2014) and performed according to the guidelines for animal experimentation by the Swedish Department of Agriculture. Time mated pregnant ewes underwent ultrasound to confirm pregnancy at least 1 week before operation. At gestational age of 94–96 days sheep underwent aseptic surgery as previously described.^16^ Prior to anesthesia induction, the ewe was given Stesolid (Diazepam, 0.1–0.2 mg/kg, i.v.). Anesthesia was induced by sodium pentothal (13 mg/kg, i.v.), followed by intubation and isoflurane (1.5%) and the ewe was also given one injection of Temgesic (Buprenorphine, 0.005–0.02 mg/kg, i.v.) and Garamycin (Gentamicin, 5 mg/kg, i.m). The uterine horn was exposed through a midline incision and a small hysterectomy incision was made over the fetal head through the uterine wall, parallel to any vessels. An inflatable silastic cuff was placed around the umbilical cord (OCHD16, DocXS Biomedical Products, Ukiah, CA). Polyvinyl catheters (i.d. 1 mm, Smiths Medical & tip 0.4 mm, Agnthos, Sweden) were inserted into each brachial/axillary artery and brachial vein. An amnion catheter (i.d. 2.0 mm, Portex, Smiths Medical, Minneapolis, MN) was secured to the ear. In case of twins, only one fetus was instrumented. At the end of the operation, catheters were filled with 50E/ml heparinised saline. The uterus was closed in two layers and catheters exteriorized via a trocar. One catheter was placed in the tarsal vein of the ewe. Sheep were allowed to recover for 3–5 days following surgery before experiments began. During this period Gentaject (Gentamicin; 5 mg/kg, i.v.) was administered to the ewe daily.

### Experimental strategy and protocol

Induction of intrauterine asphyxia was achieved by complete occlusion of the umbilical cord (UCO), by inflating the silastic cuff placed around the umbilical cord, with sterile water for 25 min in 99–101-day old fetal sheep (*n* = 10) as previously described.^16^ Fetal blood samples (60 ul) were collected prior to (−1 h) and following umbilical cord occlusion (UCO; +6 h, +24 h, +72 h, +7 days), collected into EDTA-coated tubes together with 180 ul distilled water, snap frozen and kept at −80 °C until RNA extractions. Additionally, blood sampled from the axillary catheter (50 ul) was also used for the measurement of blood gases and glucose/lactate (Radiometer ABL 725, Copenhagen, Denmark) at same times as above but also during the UCO (+20 min) and at termination (+14 days). A H_3_-antagonist^17^ was infused between day 2 to 5 (16 h per day; 1 mg/ml; rate 0.7 ml/h) after UCO to half of the animals (*n* = 5). The other fetuses received saline infusion (VEH, *n* = 5). At 14 days following UCO the experiment was terminated by an i.v. infusion of pentobarbital to the maternal vein. The fetus was immediately removed from the uterus, weighed and sexed and the fetal body, including the brain, was perfused with sterile saline, followed by 4% paraformaldehyde (Sigma-Aldrich, Germany) and remained in 4% paraformaldehyde for minimum 2 weeks, after which the brains were processed for paraffin embedding. Data of blood gases were analyzed by repeated measures ANOVA compared to pre-UCO levels using GraphPad Prism V10.0.2.

### Neuropathology assessment

Evenly spaced coronal sections (10 µm) of the forebrain were cut on a microtome as previously described.^18^ Sections were stained with acid fuchsin/thionin for morphological analysis, and adjacent sections were used for immunohistochemical analysis. Briefly, for immunohistochemical staining sections were dewaxed and rehydrated in serial decreasing concentrations of ethanol. Antigen retrieval was performed by heating the sections in 10 mM boiling sodium citrate buffer (pH 6.0) for 10 min and left to cool for 20 min, followed by blocking endogenous peroxidases with 3% H_2_O_2_ for 10 min. Nonspecific binding was blocked for 60 min with 4% horse, goat or donkey serum (same species used to raise the secondary antibody) in PBS. Sections were then incubated overnight at 4 °C with primary antibodies for either mouse anti-glial fibrillary acidic protein (GFAP, 1:250; Sigma-Aldrich, Germany), rabbit anti-ionized calcium binding adapter molecule −1 (Iba-1, 1:1000; Wako Chemicals, Germany) and rat anti-galectin-3 (anti-Mac-2, 1:100, culture supernatant from the hybridoma M3/38; LGC Promochem/ATCC; Manassas, VA). The following day, appropriate biotinylated secondary antibodies (1:250; all from Vector Laboratories, Burlingame, CA) were added for 60 min at room temperature. Visualization was performed using the Vectastain ABC Elite Kit (Vector Laboratories) with 0.5 mg/ml 3,3′-diaminobenzidine enhanced with 15 mg/ml ammonium nickel sulfate, 2 mg/ml β-D-glucose, 0.4 mg/ml ammonium chloride and 0.01 mg/ml β-glucose oxidase (all from Sigma-Aldrich).

Two anatomical levels of the brain; section 720 at the level of lateral ventricle and section 1080 at the level of the thalamus and hippocampus according to the Sheep Ovis Aries atlas (https://www.msu.edu/~brains/brains/sheep/index.html) were used for analysis. Acid-fuchsin/thionine–stained sections were examined for gross structural damage, including areas of pallor, increased cellularity, necrosis, and infarction. Astrogliosis (GFAP-positive cells), microglia activation (ameboid Iba1-positive cells and galectin-3 positive cells) was determined in the same areas in adjacent sections. The cortex, subcortical and periventricular white matter, corpus callosum, external capsule, striatum/basal ganglia, hippocampus and thalamus were all assessed. A neuropathology score was assigned to each brain region and section (according to Table 1) and summed for each animal. All neuropathology scoring was performed by an assessor blinded to the identity of the treatment group. Based on the neuropathology score the animals were divided into fetuses with mild (*n* = 4, score <10) and severe (*n* = 6, score >10) injury. For correlation analysis with transcriptome results, the neuropathology score was transformed to a categorical value and used as a measure of degree of brain injury. The H_3_-antagonist infusion did not affect brain injury scores and all animals were therefore combined in the correlation analysis.

**Table 1:** Scoring template for neuropathology based upon tissue damage (Acid-fuchsin), inflammatory response (Iba1 + Gal-3) and astrogliosis (GFAP).

Neuropathology	
Acid-Fuschin/Thionin	
0	Absent
0.5	Small lesions/extensions from other regions
1	Present: thinning/honeycomb appearance; increased cellularity; tissue breakdown
2	PVL/IVH; severe tissue loss; loss of cells
Inflammatory response	
Microglial activation/aggregations	
0	No or few signs of microglial cell activation
0.5	Mild presence of microglial aggregates/ activation
1	Moderate
2	Severe, large area of microglial aggregates’
3	Cystic white matter lesions; surrounded by/containing macrophage like IBA1^+^ cells.
Galectin-3 positive microglia (ameboid morphology)	
0	Not present
0.5	Mild
1	Moderate
2	Severe
Astrogliosis	
GFAP-positive reactive astrocytes	
0	No “reactive” astrocytes
0.5	Mild reactive astrocytes
1	Moderate reactive astrocytes
2	Severe presence of reactive astrocytes

Adapted from refs. ^39,40^. See Fig. 2 for examples of scoring.

*PVL* periventricular leukomalacia, *IVH* intraventricular hemorrhage.

### RNA extraction and expression profiling

One µg of RNA was prepared from the fetal blood and analyzed at the Genomics Core Facility, Sahlgrenska Academy, Gothenburg University and processed for RNA sequencing (RNAseq) libraries (Illumina Next Seq 500; CA). Sequencing libraries were analyzed on an Illumina HiScan, with a target read depth of ~100 M reads. Samples were demultiplexed, mapped to the reference sheep genome (ENSEMBL), and reads for known genes were counted using HTSeq.^19^ Read count data were normalized by the trimmed mean of M-values (TMM) procedure as implemented by the package edgeR. Due to low sample quality two samples were not profiled (at 72 h and 7 days respectively), the remaining samples (*n* = 48) passed both pre-alignment (analysis of sequence quality, presence of adapters and GC content) and post-alignment quality checks such as percentage of mapped reads and uniformity of read coverage on genomic region.

### Study design and time series analysis

Whole blood from ten fetal sheep was profiled across 5 different time points, −1 h, 6 h, 24 h, 72 h and 7 days after umbilical cord occlusion respectively with pre-UCO (−1 h) samples used as baseline. To perform a time series analysis of the sequencing data we used the R package maSigPro^20^ that uses a generalized linear model to model the count data. In the first step, a statistical procedure was used to identify genes with significant expression changes pre- and post-UCO using a multivariate linear regression model. The post-UCO sample group was obtained by combining the samples from time points 6 h, 24 h, 72 h and 7 days. In the second step a polynomial regression model was fitted to the data, to disclose the patterns of significant differential time evolution in a gene-by-gene fashion, and the goodness of fit, *R*^2^, of each optimized gene model was computed. This parameter, together with the corresponding corrected *p* value, was then used to select genes following trends of expression changes over time statistically consistent across all samples (*R*^2^ ≥ 0.6 and FDR < 0.05).

For this analysis, we used only the vehicle samples (*n* = 38), to avoid the potentially confounding effect of the treatment administered before 72 h to half of the animals. The identified genes were then divided into two groups based on their up- or down-regulation at time point 6 h. This is because this time point is the one closest to the induction of UCO and therefore most likely to have the highest biological relevance, and not confounded by processes related with the normal developmental stages of the fetus taking place at later time points. The two gene groups were characterized through network and pathway-based approaches to get insights into the different pathways affected by using the Ingenuity pathway analysis software (IPA). To this aim, since IPA annotation database is based on human, mouse and rat species, sheep genes were first mapped to their corresponding orthologs in humans using ENSEMBL Biomart tool.

### Associating gene expression levels with brain injury

To identify genes whose expression in blood may be associated with the severe vs. mild brain injury phenotype, we ran a multi-variate linear regression model, using data at each time-point separately. RNA-Seq count data were first transformed using the voom function^21^ and then processed using the R package LIMMA to model the mRNA expression levels as a function of degree of brain injury and accounting for treatment:

$$\,{{{{{{\rm{lmFit}}}}}}}({{{{{{\rm{expression}}}}}}} \sim {{{{{{\rm{brainInjury}}}}}}}+{{{{{{\rm{treatment}}}}}}})$$

### Analysis of blood composition from RNA-Seq data

In order to infer how blood composition changes across time, we developed a novel analytical procedure based on CIBERSORT, a recently published and highly validated method for the inference of cell-type abundances in mixed cell/tissue populations.^22^ The method implements linear support vector regression and uses a signature expression matrix to infer abundances of leukocyte sub-populations. This leukocyte gene signature matrix, termed LM22 comprises 547 genes that distinguishes 22 human hematopoietic cell phenotypes, including naïve and memory B cells, seven T-cell types, natural killer cells, plasma cells and myeloid subsets. CIBERSORT was originally developed by Newman and colleagues for the deconvolution of microarray data sets in humans, therefore we could not directly employ its deconvolution algorithm to our fetal sheep RNA-Seq data. However, since the cell type markers (LM22 signature) was successfully employed to estimate the relative cell frequencies from the gene expression data we used these to characterize the cell type differences between the pre- and post- asphyxia samples by using a different algorithm than Newman et al.^22^ First from the original list of 547 leukocyte genes, we selected the markers specific to each of the cell-type (Supplementary Table 1 of Newman et al.). Then we sub-selected the genes having a close orthologue in sheep, leaving us with a list of 165 genes. Each sample was then ranked based on the gene expression of each of these cell-type specific genes (Supplementary Fig. 1). Sample rankings obtained for each cell type specific gene were then aggregated by taking the sample median for each cell type (e.g., B-cells, T-cells etc.). In this way we could assess the extent to which a cell-type was represented in each sample, with respect to all other samples. These ranks were used to run comparative analysis between the pre- (*n* = 10) and the post-UCO samples (*n* = 28, from time points 6 h, 24 h, 72 h, 7 days) using the Wilcoxon rank sum test. Results expressed as Wilcoxon *U*-statistics values were compared with those obtained from the same data after randomly assigning labels to the samples (permutation without replacement, *N* = 1000). The number of times the *U* statistic from the randomized runs was lower than the real data was recorded. Wilcoxon analysis results were also compared with those obtained from LIMMA multivariate modeling, where log transformed rank scores were expressed as a function of injury and time and subject-specific effects were considered as a random effect. Both randomization procedure and LIMMA modeling results showed complete convergence with standard Wilcoxon analysis on real data both in terms of *p* values and, for LIMMA, of fold-changes.

## Results

### Blood gas analysis and brain injury

**Fig. 1:**
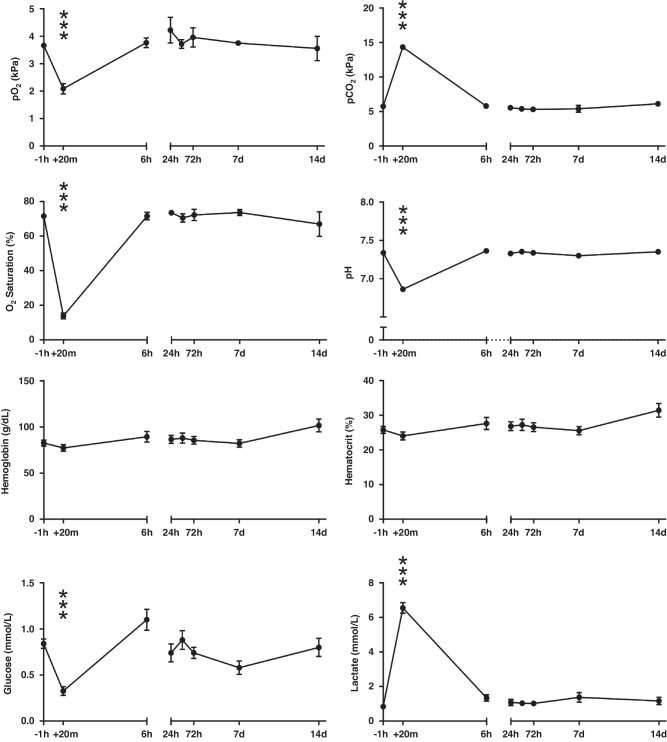
Blood analysis before and after umbilical cord occlusion (UCO). Blood gases, hemoglobin, hematocrit, glucose and lactate levels in pre- (−1 h), during (+20 min) and post-UCO blood samples (+6 h, +24 h, +72 h, +7 days and +14 days). *n* = 7–10 per time-point. Data are mean ± SD, repeated measures ANOVA (compared to pre-UCO levels), ***p < 0.001.

Following occlusion there was a rapid (20 min) drop in pO_2_ (−44%), O_2_ saturation (−78%), pH (−6%), and glucose (−64%) while there was an increase in pCO_2_ (+22%) and lactate (+443%), see Fig. 1 (*p* < 0.001 across all). Hemoglobin and hematocrit levels were not altered at any time-point (*p* > 0.05). Data analyzed by repeated measures ANOVA compared to pre-UCO levels. All parameters from the blood gas analysis normalized by 6 h (i.e., similar to pre-UCO levels; *p* > 0.05) and remained this way until 14 days after the occlusion when animals were terminated. Regional neuropathology scores for each animal are summarized in Table 2 along with median values in data. Overall pathology was mainly localized to white matter regions, with highest injury scores for the periventricular and intragyral white matter (median score 3.5 and 5, respectively), somewhat less to the corpus callosum (median score 1.25) but also in the basal ganglia and thalamus (median score 2). Minimal pathology was observed in cortical gray matter, hippocampus, cerebellum and none in the brain stem. One animal showed presence of intraventricular hemorrhage and also showed the highest neuropathology score.

**Table 2:**
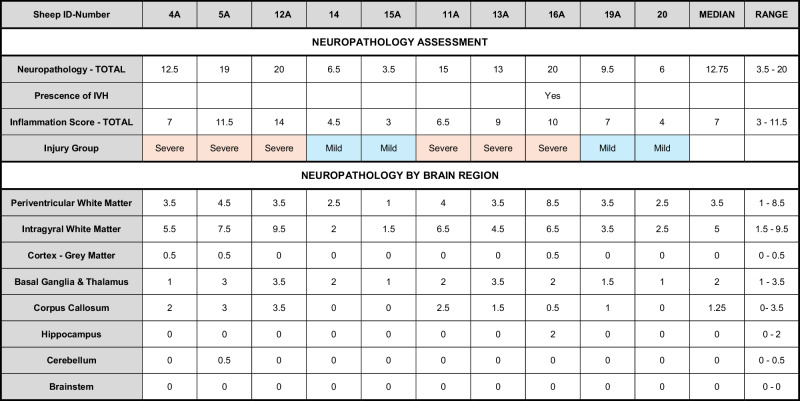
The brain injury score for each brain region in all animals.

From this score animals were then divided into two injury groups (Mild = Total neuropathology score <10 (blue); Severe = score >10 (red)). Thus, sheep-ID 14, 15A, 19A, 20 were classified as mild and sheep-ID 4A, 5A, 12A, 11A, 13A, 16A as severe. The total neuropathology score is the sum from all assessments outlined in Table 1, while total neuroinflammation score is only the inflammatory response score as defined in Table 1.

### Genes perturbed with injury over time

**Fig. 2:**
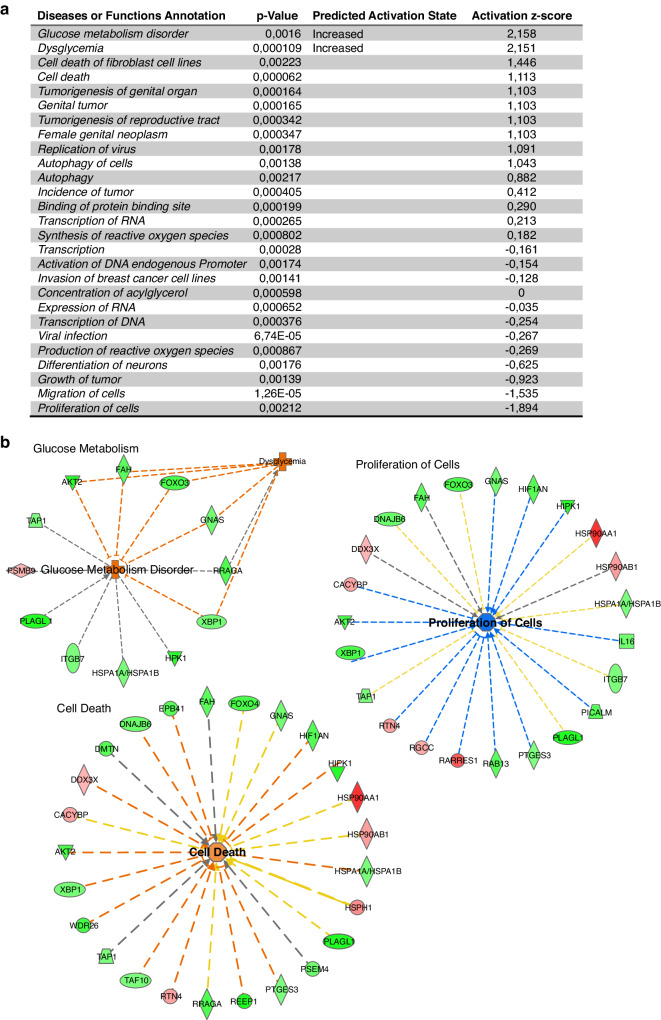
Pathway analysis of gene changes after UCO. **a** Downstream effect analysis using IPA of post-UCO regulated genes listing all biofunctions with *p* < 0.05. Predicted increase of glucose metabolism disorder and dysglycemia (*z*-score = +2.16 and +2.15, respectively). Cell death shows second highest *z*-score (+1.11) while proliferation of cells shows highest negative *z*-score (−1.86). **b** Networks of molecules related to dysglycemia, cell death and proliferation of cells.

**Fig. 3:**
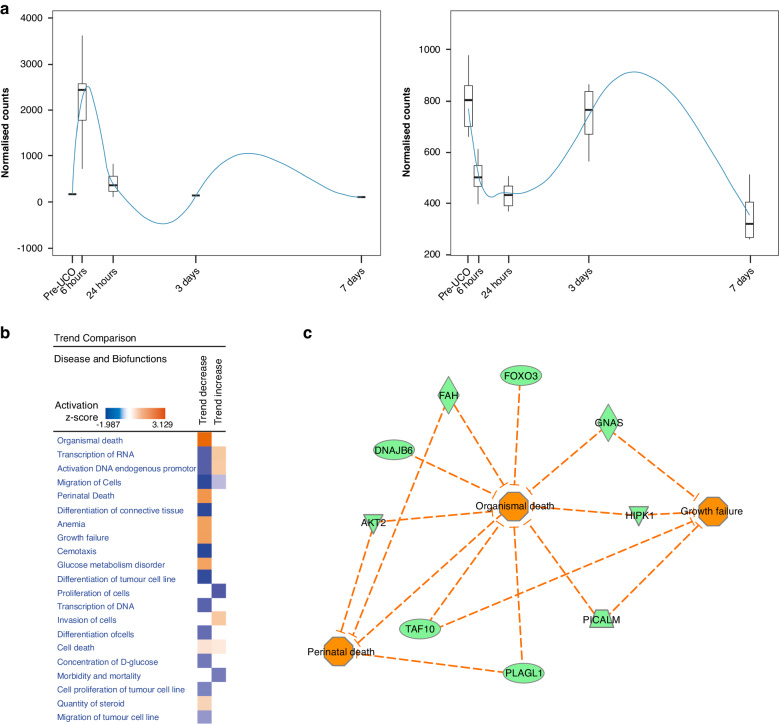
Trend analysis of pre/post-UCO genes. **a** Polynomial regression models for pre-/post-UCO regulated genes. **b** Trends comparison (using IPA) of down-stream effect analysis generated from genes up- or down-regulated at 6 h post UCO. **c** Molecular network for perinatal death, organismal death and growth failure.

To characterize the gene expression changes following asphyxia we performed a trend analysis on the vehicle samples (Total *n* = 40: pre-UCO *n* = 10; 6 h *n* = 10; 24 h *n* = 10; 3-days *n* = 5; 7-days *n* = 5) across the five-different time-points (pre- and post-UCO), using the maSigPro R Bioconductor package.^20^ Thereby 61 genes were identified with statistically significant altered expression trends, consistent across all samples (Supplementary Table 1). IPA downstream effect analysis using all genes with altered expression trends resulted in significant positive scores (*z*-score >2, thus predicting increase) for glucose metabolism disorder (+2.16) and dysglycemia (+2.15) followed by cell death (+1.11) whereas greatest negative *z*-score (predicting decrease) calculated for cell proliferation (−1.89). Full list of all biofunctional annotations with significant *p* value can be found in Fig. 2 along with networks to above functions. These genes were then divided into two groups, based on their up- (*n* = 25) or down-regulation (*n* = 36) at the 6 h time point. The rationale for choosing the 6 h time-point was that gene expression at later time-points (24 h, 72 h and 7 days) was more likely to be influenced by normal developmental processes and therefore may not be fully related to the reaction to injury. We then carried out a comparative analysis using the IPA pathway analysis software to highlight the biological pathways and networks affected by these expression changes, in both directions (Fig. 3b). Interestingly, among the genes down-regulated at 6 h (immediately after injury), *Sox6, Plagl1, Gnas, Fah* and *Akt2*, were identified to play a role in perinatal death. Others, are known to activate growth failure processes (*Gnas, Hipk1, Picalm* and *Taf10*) and organismal death (*Sox6, Plagl1, Gnas, Fah*, *Akt2, Dnajb6, Taf10, Picalm, Hipk1, Foxo3*), see Fig. 3.

### Blood gene expression changes at Pre-UCO and 6 h as a biomarker of brain injury

**Fig. 4:**
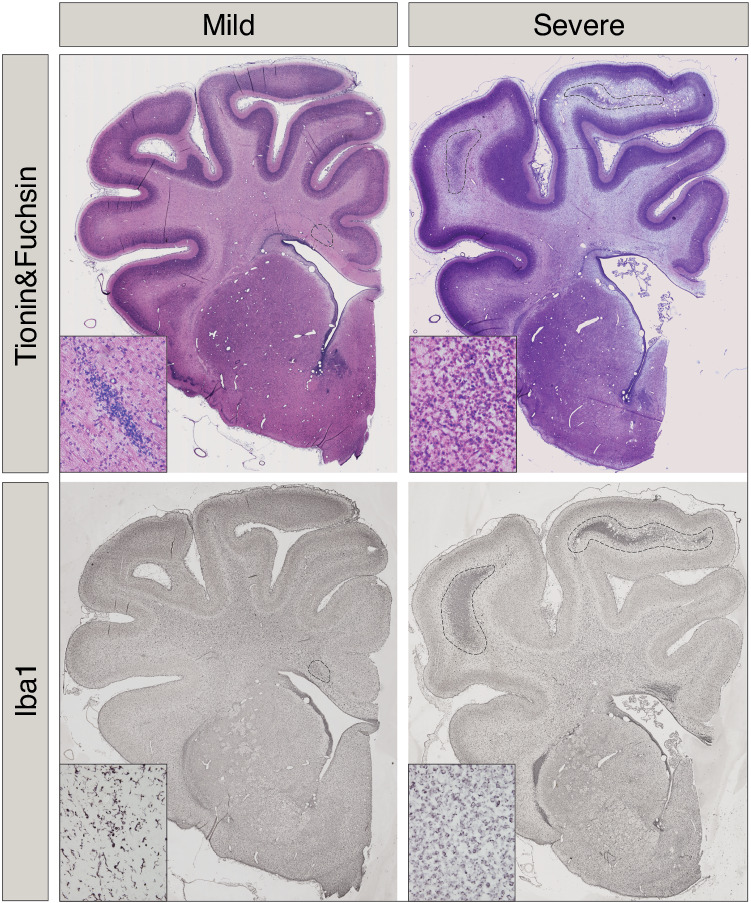
Brain sections, at the level of the lateral ventricle, were stained with acid fuchsin/thionine or Iba-1 (microglia marker). In the severe injury group note regions in the subcortical white matter with pyknotic cells and microgliosis (dashed areas), see also Table 1.

**Fig. 5:**
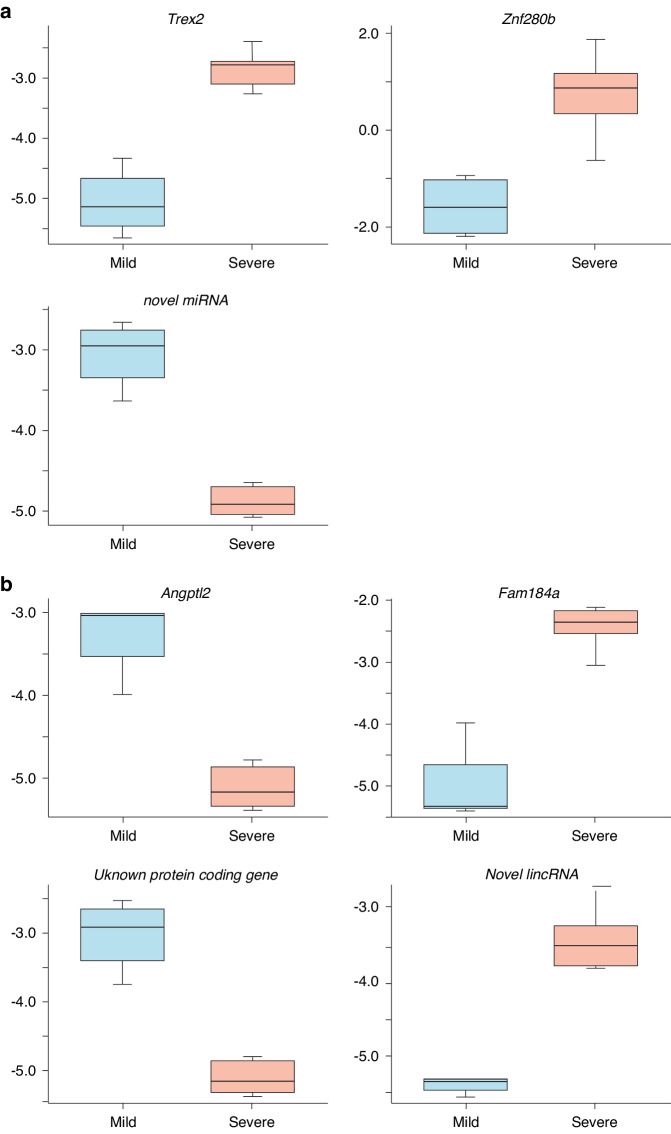
Genes associated with mild or severe injury. **a** Pre-UCO gene levels in blood significantly associated with mild or severe brain injury. **b** Six hour post-UCO gene levels in blood associated to mild or severe brain injury. FDR < 0.01 was used to identify these genes.

Next analysis was aimed at exploring the association between gene expression at early time points—pre-UCO and 6 h after UCO—with the binary mild vs. severe brain injury phenotype. Injuries were determined using an array of injury assessments with examples of Acid Fuchsin/Thionin staining (Neuropathology) and Iba1 immunohistochemistry (microglia activation) in mild vs. severe injuries illustrated in Fig. 4. In pre- UCO samples, 3 genes were found to be significantly different (Fig. 3) between severely and mildly injured samples (FDR < 0.01). These were *Trex2*, *Znf280b* and an unknown novel miRNA (ENSOARG00000024513). This time-point is particularly interesting as it points to the existence of a different gene expression pattern before UCO in sheep who will develop mild vs. severe injuries. The *Trex2* (three prime repair Exonuclease 2) gene is involved in DNA replication, repair and recombination and was considerably downregulated in mildly injured sheep. By analyzing data from samples at 6 h after asphyxia, 4 genes, *Angptl2, Fam184a*, *Novel lincRNA* (ENSOARG00000026669) and an unknown protein coding gene (ENSOARG00000020183) were also identified to be associated with the degree of injury with an FDR < 0.1 (Fig. 5).

### Significant changes in blood immune composition between pre- and post-UCO samples

**Fig. 6:**
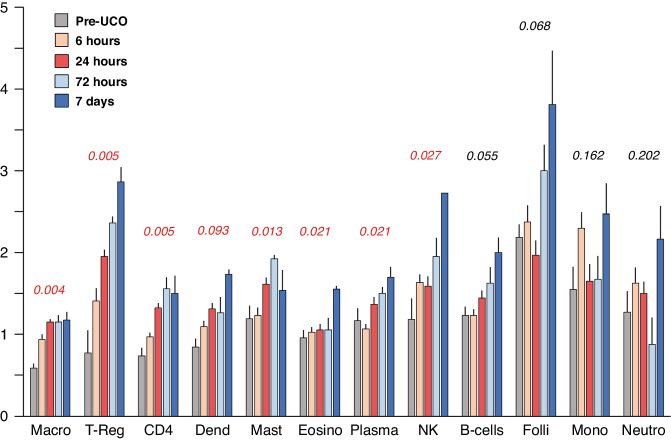
Changes in blood cell composition inferred by RNAseq expression profile post-UCO using validated leukocyte markers (CIBERSORT). Numbers indicate *q*-values.

To shed light on the activation of immune system components resulting from the induction of asphyxia and the consequent tissue damage, we investigated the changes in blood composition, as inferred from RNA-Seq gene expression data. To this aim we developed a unique ranking method largely based on previously developed leukocyte markers from CIBERSORT.^22^ Briefly, it was different from CIBERSORT as (1) only cell-type specific genes, having an orthologue in sheep were used to infer cell-type abundances (2) comparative results were obtained from non-parametric analysis of aggregated gene rankings. The validity of our ranking strategy was comparatively assessed with randomization procedure and multivariate regression alternative approaches, demonstrating full convergence of the results (see “Methods”). By using this approach, we observed that macrophages, T regulatory cells, CD4 cells, dendritic cells, mast cells, eosinophils, plasma cells and natural killer cells undergo statistically significant changes (*q*-value < 0.05) after UCO (Fig. 6).

## Discussion

Predictive blood biomarkers for neonatal brain injuries could be beneficial on their own or in conjugation with other predictive prognostic tools, such as neuroimaging and EEG. With this in mind, we determined whole transcriptomic mRNA levels in blood before and after asphyxia using a clinically relevant preterm sheep UCO model and found early novel candidate biomarkers associated to brain injury. For broad-spectrum brain injury scores, we used an array of assessments including neuropathology and cellular responses in brain tissue sections 2 weeks after UCO. We found that 61 blood mRNAs were changed after UCO, with a smaller number of mRNAs before and after induction of asphyxia corelated to injury outcome. Moreover, we successfully developed a ranking-based method that can be used to estimate blood cell composition from the sheep blood transcriptome expression profiling that predicted significant changes in blood cell composition following UCO.

### Candidate biomarker mRNAs of brain injury

The main aim of this study was to find early blood biomarkers that correlate to brain injury outcome. We found seven pre-/post-UCO mRNA transcripts that were associated with outcome and hence might serve as potential biomarkers. Four of these transcripts were protein-coding, two miRNAs and one linkRNA. At this stage we can only speculate whether changes in these genes are causal to injury, or an association exist because of secondary events, in either case these mRNAs could potentially serve as biomarkers. That we found pre-UCO mRNAs that were associated to outcome is noteworthy since it points to that potentially there is a genomic predisposition to these injuries. The three pre-UCO transcripts that were associated to outcome were *Trex2*, *Znf280b* and one novel miRNA. TREX2 (Three prime repair exonuclease 2) is normally expressed in squamous epithelia such as in keratinocytes and esophagus but its exact biological functions are still unclear. The *Trex2* gene encodes for an enzyme with exonuclease activity, such enzymes are normally involved in DNA replication and repair, but TREX2 appears to both alter and maintain genome stability, playing a role in DNA-repair.^23^ This particular exonuclease is best known for a role in skin repair after DNA damage.^24^ Supporting a role of this gene for injury progression is that *Trex2* deficient mice have a milder inflammatory response to induced psoriasis-like phenotype, with down-regulation of genes related to immune cell chemotaxis, cell killing, interferon responses and apoptosis.^24^

ZNF280B, which had higher blood mRNA levels found in sheep with severe injuries, is a zinc finger protein and shown to upregulate oncogene MDM2 (an ubiquitin ligase) which in turn negatively regulates tumor repressor p53 which previously has been implied in neonatal brain injury.^25^ There was also one novel miRNA pre-UCO associated to outcome. Although the sequence shows high homology to Hsa-mir-5703 (98%), this miR has no validated targets to date.

Four mRNAs that were regulated at 6 h post-UCO were also associated to outcome and thus are potential prognostic biomarkers. *Angptl2* (Angiopoietin-like protein 2) was markedly down-regulated at 6 h post-UCO in severely injured sheep. ANGPTL2 is a multifaceted secreted glycoprotein acting on endothelial cells promoting angiogenesis but has also been shown to have roles in inflammation and tumor expansion. In general, promoting vascular regeneration has been shown to be beneficial in different models of ischemia and VEGF, a potent angiogenic protein, has been shown to be neuroprotective in experimental model of neonatal hypoxia-ischemia (HI).^26^ Moreover, in studies it was found that after birth asphyxia in humans, babies with pro-angiogenic factors in the blood have better outcome.^27^ In addition, adult stroke studies in mice have shown that macrophage derived ANGPTL2 promotes neuroinflammation and result in more severe brain injury.^28^ Our results are in line with these studies and underscores the importance of vascular responses in injury progression. High levels of *Fam184a* were associated with severe injuries, however, the functional knowledge of FAM184A is limited. It appears to be a domain of CEP164 in humans which regulates cilia formation and in vitro studies have shown that it maintains genomic stability in relation to UV-light induced DNA damage.^29^ It has been identified as a risk gene in endometrial cancer^30^ and predicted to be a major influencer gene in chronic graft vs. host disease after allogeneic hematopoietic stem cell transplantation.^31^ We also found one lincRNA (Long intergenic non-coding RNA) and an unknown protein coding gene that correlated to outcome. The unknown protein has an Ina-F motif, a highly conserved motif that is thought to be a transmembrane helix that binds to transient receptor potential (TRP) calcium channel. TRP-related channels have shown to mediate store-operated calcium entry, important for Ca^2+^ homeostasis in a wide variety of cell types. We cannot speculate much about the lincRNA interactions although its best match in the human genome is H1FX, a histone H1 family protein. Taken together, with the present untargeted approach we have identified several novel biomarkers of fetal asphyxia related brain injury in preterm sheep fetuses. From previous studies it is clear that some of these genes are related to functions that we know are important in HI-related brain injury, such as angiogenesis and inflammation, and thus are likely involved in injury processes or related to potentially important functions such as genome repair and stability. However, since several other genes are novel or have unknown function at this stage, further functional annotation of these genes and mRNA transcripts in relation to ischemic injury would be of great interest and additional validation as biomarkers is needed to test their robustness in different injury models and in the clinics.

### Functional annotation and regulated genes related to fetal growth, development and cell death

In support of our results, down-stream effect analysis in IPA of post-UCO regulated genes predicted dysglycemia after UCO. As has been shown previously in birth asphyxia models in sheep, we found marked hypoglycaemia following UCO (Fig. 1). Similarly, hypoglycaemia is one of the common manifestations after asphyxia in newborns. Further, the candidate genes emerging from our trends pre-/post-UCO time series analysis are known to play a role in fetal growth and development (see Fig. 2). As an example, *Plagl1*, which was down-regulated at 6 h post-UCO, is a maternally imprinted gene and a master regulator of embryonic growth. Loss in methylation of PLAGL1 has been linked with developmental disorders such as intrauterine growth restriction^32^ whereas increases in methylation of PLAGL1 has been associated with chorioamnionitis and funisitis in studies on preterm birth.^33^ Vincent et al. concluded that *Plagl1* is a key gene during protection against ischemia-reperfusion injury in heart.^34^ Similarly, deficiency of *Picalm*, another gene which was down-regulated at 6 h post-UCO, was shown to result in growth retardation and defects in mice in utero.^35^ Another gene regulated after UCO, *Sox6*, encodes for a transcriptional activator that is required for normal development of the central nervous system, chondrogenesis and maintenance of cardiac and skeletal muscle cells.^36^ In vitro studies using Sox6-antisense indicate that Sox6 plays an important role in neuronal differentiation and apoptosis.^37^ *Foxo3*, which was downregulated in our model of UCO, belongs to a group of FoxO-transcription factors that are important in antioxidant defences and also regulates apoptosis. Deletion of FOX03a was shown to results in limited differentiation of neuronal stem cells.^38^ At the same time, FOXO3 phosphorylation and subsequent translocation to the nucleus is a critical step in inducing neuronal apoptosis in neonatal HI in rats.^39^ *Hipk1*, down-regulated post-UCO, encodes for protein kinase which has been shown to be control vasculogenesis thereby loss of *hipk1* and *hipk2* upregulates pro-angiogenic genes, such as *mmp10* and *vegf*, resulting in endothelial proliferation.^40^ *Akt2* that encodes for a RAC-beta serine/threonine-protein kinase and is related to survival responses was down-regulated post-UCO. While Akt1 and 3 have been shown to be downregulated following stroke and overexpression is neuroprotective in an mTOR-dependant manner^41^ to our knowledge Akt2 has not previously been shown to change after brain injury. There are several studies showing an important role of AKT2 in cardiac injury. Ablation of *akt2* was protective against LPS-induced cardiac dysfunction^42^ as well as implicated in the regulation of cardiomyocyte metabolism and survival. *Akt2* deficient mice have also been found to be more prone to cardiomyocyte apoptosis, with apoptosis significantly increased in the peri-infarct zone in hearts of Akt2-ko animals after cardio-ischemic injury.^43^

### Cell profiling in blood from whole transcriptome data

We adapted a recently developed algorithm to assess different cell types in complex tissue composition from RNA-Seq gene expression data^22^ to study blood cell composition. Using this method, we detected significant changes in markers for several blood cell types in response to fetal asphyxia in the sheep. A limitation of the present study is that we cannot predict from the gene analysis whether the changes are due to altered gene activity, or whether there has been a change in the relative abundance of cells in which the gene is expressed. A previous study described an increase in total white blood cell counts 1 day after UCO in fetal sheep, while flow cytometry analysis did not reveal any differences in neutrophils, helper T-cells or cytotoxic T-cells.^44^ Leukocytes are the main chromosome containing cell type in adult blood, however, in the fetus and newborn up to 10% of nucleated cells consist of nucleated red blood cells.^45^ Thus, our analysis presumably represents mainly the biological state of white blood cells. In support, we identified changes in different leukocyte subtypes, such as macrophages, T regulatory cells, CD4 cells, dendritic cells, mast cells, eosinophils, plasma cells and natural killer cells. The contribution of peripheral immune cells in perinatal brain injury remains largely unknown. Previous studies suggest that depletion of neutrophils prior to neonatal HI can reduce injury.^46^ Our recent study in neonatal mice, demonstrated that peripheral immune cells enter the brain after HI and that blocking monocyte and neutrophil trafficking after HI was neuroprotective in males.^47^

### Limitations of study

There are a number of considerations/limitations of study that should be mentioned. As discussed above, the etiology behind brain injury in preterm infants is complex and multifactorial including inflammation/infection, hypoxia, ischemia, hyperoxia, hemorrhage and the predominant etiology varies from case to case. We believe that hypoxia model represents one of these etiologies and is therefore of clinical relevance but does not model the whole spectrum of underlying injury mechanisms. Thus, the mRNAs associated with worse outcome that we have identified in our study should be tested in other brain injury models with different etiology as well as in the clinical setting. This is particularly important since the present study involves a limited number of animals. Lastly, our study design is a before and after approach which has its strength in that animals are followed over time, however, there are also natural developmental aspects that could have an influence which is difficult to correct for without a non-asphyxiated control group.

## Conclusions

We have used a whole transcriptomic approach together with a comprehensive neuropathology assessment in order to uncover novel biomarkers in blood for UCO-induced brain injury in the preterm sheep brain. This approach may also lead to new insights in the injury development. Interestingly, we found mRNAs in blood both pre-UCO and post-UCO that were associated to injury. The former thus indicates that levels of these mRNAs underlies susceptibility to the injury and could have genetic origin. Some of these mRNAs have functions that are likely to play a role in pathology processes such as angiogenesis, inflammation and DNA repair while others, in particular several non-coding RNAs, have unknown function at this point. It would be of great interest to further define the role of these mRNAs in the injury progression. Although we have used a large animal model which closely replicates several key aspects of brain injury in the preterm the results need to be further validated in other animal models and various longer-term outcomes in order to be translated into the clinic. These should also be tested along with other parameters predictive of injury to strengthen the prognostic resolution.

## Supplementary information

Supplementary table and figure

## Data Availability

The data that support the findings of this study are available from the corresponding author upon reasonable request.
