# Supplementary material for: Novel biomarkers of preterm brain injury from blood transcriptome in sheep model of intrauterine asphyxia
Source: Pediatr Res. 2024 May 31;96(7):1707–17. doi: 10.1038/s41390-024-03224-1 (PMC11772238; doi:10.1038/s41390-024-03224-1)
Supplement: Supplementary file 1 — Supplementary table and figure [file 41390_2024_3224_MOESM1_ESM.pdf]

Suppl. Table 1. Genes identified with statistically altered expression across all time-points (pre- and post-UCO).

Entrez Gene Name	Symbol	Fold Change
acyl-CoA thioesterase 7	ACOT7	1.155
ARP1 actin-related protein 1 homolog A, centractin alpha	ACTR1A	-1.082
AKT serine/threonine kinase 2	AKT2	-1.675
calcyclin binding protein	CACYBP	1.229
chromodomain helicase DNA binding protein 9	CHD9	1.106
DDB1 and CUL4 associated factor 8	DCAF8	-1.414
DEAD-box helicase 3, X-linked	DDX3X	1.028
dematin actin binding protein	DMTN	-1.334
DnaJ heat shock protein family (Hsp40) member A4	DNAJA4	1.151
DnaJ heat shock protein family (Hsp40) member B6	DNAJB6	-1.153
erythrocyte membrane protein band 4.1	EPB41	-1.313
fumarylacetoacetate hydrolase	FAH	-1.286
family with sequence similarity 213 member A	FAM213A	-1.307
forkhead box O3	FOXO3	-1.429
FXYD domain containing ion transport regulator 3	FXYD3	-1.262
GNAS complex locus	GNAS	-1.107
hemoglobin subunit mu	HBM	-1.389
hemoglobin subunit theta 1	HBQ1	-1.61
hypoxia inducible factor 1 alpha subunit inhibitor	HIF1AN	-1.343
homeodomain interacting protein kinase 1	HIPK1	-1.687
heat shock protein 90 alpha family class A member 1	HSP90AA1	2.858
heat shock protein 90 alpha family class B member 1	HSP90AB1	1.349
heat shock protein family A (Hsp70) member 1A	HSPA1A/HSPA1B	-1.046
heat shock protein family H (Hsp110) member 1	HSPH1	1.579
IK cytokine, down-regulator of HLA II	IK	-1.277
interleukin 16	IL16	-1.116
iron-sulfur cluster assembly 1	ISCA1	-1.217
integrin subunit beta 7	ITGB7	-1.02
kinesin family member 21B	KIF21B	1.015
meiosis regulator and mRNA stability factor 1	MARF1	-1.147
mediator complex subunit 13 like	MED13L	1.086
nuclear receptor coactivator 7	NCOA7	-1.024
OTU deubiquitinase 5	OTUD5	-1.419
phosphatidylinositol binding clathrin assembly protein	PICALM	-1.323
PLAG1 like zinc finger 1	PLAGL1	-1.716
proteasome subunit beta 9	PSMB9	1.006
proteasome activator subunit 4	PSME4	-1.137
prostaglandin E synthase 3	PTGES3	-1.006
RAB13, member RAS oncogene family	RAB13	-1.296
retinoic acid receptor responder 1	RARRES1	2.315
receptor accessory protein 1	REEP1	-1.755
regulator of cell cycle	RGCC	1.146
Ras related GTP binding A	RRAGA	-1.378
arginine and serine rich protein 1	RSRP1	1.225
reticulon 4	RTN4	1.276
small Cajal body-specific RNA 10	SCARNA10	1.248
SERPINE1 mRNA binding protein 1	SERBP1	-1.326

SH3 domain binding protein 1	SH3BP1	-1.254
SRY-box 6	SOX6	-1.11
SP100 nuclear antigen	SP100	-1.002
TATA-box binding protein associated factor 10	TAF10	-1.024
transporter 1, ATP binding cassette subfamily B member	TAP1	-1.023
ubiquitin conjugating enzyme E2 R2	UBE2R2	-1.52
ubiquitin specific peptidase 6	USP6	-1.12
WD repeat domain 26	WDR26	-1.463
X-box binding protein 1	XBP1	-1.075
zinc finger, matrin-type 2	ZMAT2	-1.189
zinc finger protein 644	ZNF644	1.175

	Gene A	Gene B	Gene C	Rank A	Rank B	Rank C	Cumulative Median Rank	Normalised Rank *(Cumulative Median Rank/No. of genes)*
Sample 1	5.28	24.14	7.35	4	5	5	5	5/3= 1.67
Sample 2	3.33	17.19	6.23	3	3	4	3	3/3=1.00
Sample 3	2.69	15.87	3.82	2	2	1	2	2/3=0.67
Sample 4	5.42	22.17	5.36	5	4	3	4	4/3=1.33
Sample 5	1.57	6.34	4.09	1	1	2	1	1/3=0.33

Suppl. Figure 1. Ranking scheme for each sample based upon cell-type specific gene expression. The median accumulated sample ranking was used to infer cell-type specific representation for each sample (see also Figure 6).
